# Broad Antibacterial Activity and Mechanism of Garlic (*Allium sativum* L. cv. Uiseong) Extracts against Cell Wall of *Aeromonas hydrophila*

**DOI:** 10.4014/jmb.2410.10035

**Published:** 2025-02-24

**Authors:** Putri Christy Artawinata, Youjin Kim, In Young Choi, Mi-Kyung Park

**Affiliations:** 1 School of Food Science and Biotechnology and Food and Bio-Industry Research Institute, Kyungpook National University, Daegu 41566, Republic of Korea; 2 Department of Food Science, University of Wisconsin-Madison, Madison, Wisconsin 53706, USA

**Keywords:** Uiseong garlic, *A. hydrophila*, broad antibacterial activity, protein leakage, bacterial cell membrane disruptions

## Abstract

*Aeromonas hydrophila* is a pathogenic bacterium known for its resistance to antibiotics and its ability to cause infections in aquatic environments. This has made disease management more complex, making the development of alternative antimicrobial agents necessary. Uiseong garlic is a superior variety from Republic of Korea, renowned for its high level of beneficial compounds, making it a promising candidate for natural antimicrobial application. Therefore, this study aimed to investigate the antibacterial activity and mechanism of Uiseong garlic extracts against the cell wall of *A. hydrophila*. Uiseong garlic extracts were prepared using water and ethanol at 22°C and 90°C, respectively. The antibacterial activities of Uiseong garlic extracts were evaluated for their yield, antibacterial dynamics, leakage of bacterial intracellular proteins, and changes in morphological characteristics. Uiseong garlic extracts at 22°C exhibited significant antibacterial activities against foodborne pathogens, particularly against 9 strains of *A. hydrophila*. In this study, the ethanol extract at 22°C demonstrated significantly higher antibacterial activity compared to the water extract at 22°C, with a similar pattern of antimicrobial dynamics with polymyxin B. The ethanol extract at 22°C caused a higher concentration of leaked bacterial proteins (92.87±0.46 μg/ml), indicating cell membrane disruption. Additionally, transmission electron microscopy analysis further confirmed that both extracts induced plasmolysis, leading to notable damage to the bacterial cell membrane. Therefore, ethanol extract of Uiseong garlic was demonstrated as a promising alternative to antibiotics for controlling *A. hydrophila*.

## Introduction

*Aeromonas* spp. are Gram-negative, rod-shaped, motile, facultative anaerobic, and non-spore-forming bacteria [1]. It can grow at ambient temperature and are commonly found in aquatic environments, including freshwater, estuarine, and saltwater environments [2-4]. Among *Aeromonas* spp., *A. hydrophila* is a recognized organism associated primarily with fish, water, and humans [5, 6]. *A. hydrophila* is responsible for the majority (>85%) of human infections due to its ability to produce enterotoxins or cytotoxins, leading to diarrhea, nausea, vomiting, and septicemia [7-9]. Recently, *A. hydrophila* has gained attention as an emerging foodborne pathogen due to its rising outbreaks and multidrug resistance [10, 11]. As a result, *A. hydrophila* infections are becoming more difficult to manage, highlighting the urgent need for new and effective antimicrobial agents.

Phytotherapy has become more popular for controlling foodborne pathogens [12]. Phytotherapy offers an accessible, cost-effective, and eco-friendly approach to sustainable aquaculture, providing a natural alternative to synthetic pharmaceuticals in reducing pathogens [8]. Garlic (*Allium sativum* L.) is one of the most commonly used medicinal plants included in fish diets as extracts without causing adverse side effects [8]. As a natural and safe antimicrobial agent, garlic holds great potential as a substitute for antibiotics in aquaculture. *Allium sativum* L. cv Uiseong is a domestic variety in Republic of Korea, thriving in cold weather at high latitudes, that is renowned for its robust flavor, solid tissues, and excellent storage stability [13-15]. Uiseong garlic typically consists of 6 to 7 cloves and are known for their superior appearance compared to other varieties. A previous study by Yoo *et al*. [14] highlighted that Uiseong garlic contains higher amounts of organosulfur compounds compared to other varieties in Korea that is potentially related to its antimicrobial properties [14].

Several studies have evaluated the antibacterial activity of garlic aqueous extract and ethanolic extract. These extracts of garlic have shown widespread antibacterial activities against major foodborne pathogens, including *Escherichia coli*, *Salmonella* spp., *Staphylococcus aureus*, *Bacillus* spp., *Pseudomonas aeruginosa*, *Klebsiella pneumoniae*, and *Helicobacter pylori* [16, 17]. Despite the well-documented antimicrobial properties of garlic, there are no specific studies on the antimicrobial properties of Uiseong garlic and its mechanism of action, particularly against *A. hydrophila*. Therefore, this study aimed to investigate the antimicrobial activities of Uiseong garlic extract against major foodborne pathogens, and evaluate their effectiveness in targeting cell membranes by comparison with commercial antibiotics (polymyxin B and ampicillin) against *A. hydrophila*, highlighting their potential use as natural antimicrobial agents.

## Materials and Methods

### Preparation of Garlic Extracts

Garlic (*Allium sativum*) cv. Uiseong was purchased from Uiseong, Korea. The garlic cloves were cleaned, peeled, and chopped before extraction. Extraction was carried out using filtered water and ethanol (99.9%, Duksan Pure Chemicals, Republic of Korea) as solvents at 22°C and 90°C for 12 h, respectively. The extract was filtered through filter paper (Whatman No. 4), and the filtrate was concentrated using a rotary vacuum evaporator (Ika Co., Germany) at 40°C. Afterward, the concentrated samples were freeze-dried, and the yield (%) of each extract was calculated using the following equation [18].

$$\text{Extract yield (\%)}=\frac{\text{Extract garlic powder weight (g)}}{\text{Initial garlic weight (g)}}\times 100$$

### Bacterial Strains and Growth Condition

Nine strains of *A. hydrophila* and thirteen strains of non-*Aeromonas* strains (Table 2) were provided by the College of Veterinary Medicine at Seoul National University (Korea) and the American Type Culture Collection (ATCC). Each bacterium was grown in 25 ml of tryptic soy broth (TSB, Difco Laboratories Inc., USA) at 37°C with shaking at 110 rpm for 16 h. The overnight culture was washed three times with sterilized phosphate-buffered saline (PBS, pH 7.4, Life Technologies Co., UK) and centrifuged at 7,000 ×*g* for 4 min to obtain bacterial cells. The concentration of each bacterial suspension was adjusted to 10^8^ CFU/ml by using a preconstructed standard curve measured at the optical density of 640 nm [19].

### Antibacterial Activities of Uiseong Garlic Extracts

The antibacterial activity of garlic extract against 22 strains of foodborne pathogens, including *Aeromonas* spp. was determined using a disc diffusion method [20]. The mixture of 1 ml of each bacterial suspension (10^8^ CFU/ml) and 10 ml of molten tryptic soy agar (TSA, Difco Laboratories Inc.) was poured onto the plate immediately. Fifty microliters of garlic extract (500 mg/ml) were impregnated into a sterilized filter paper disc (8 mm, Advantec Co., Japan). Ampicillin and polymyxin B (1 mg/ml Sigma-Aldrich Co., USA) were used as positive controls, while distilled water was used as a negative control. The impregnated disc was placed on the surface of a solidified TSA plate and incubated at 37°C for 18 h. The antibacterial activity of each garlic extract was expressed by measuring the diameter (mm) of the clear zone.

### Minimum inhibitory concentration of Uiseong garlic extracts

The minimum inhibitory concentration (MIC) test of each Uiseong garlic extract against nine strains of *A. hydrophila* (Table 3) was conducted using a standard Bauer-Kirby disc diffusion method with minor modifications [21]. Each serially diluted Uiseong garlic extract (500, 400, 300, 200, and 100 mg/ml) was impregnated to the disc and then placed on the Mueller-Hinton agar (MHA) surface. The plates were then incubated at 37°C for 18 h. The lowest concentration for inhibiting bacterial growth was determined to be the MIC value [22].

### Antibacterial Dynamics of Uiseong Garlic Extracts

The evaluation of the antibacterial dynamic was conducted using a liquid culture inhibition assay [23]. 500 μl of bacterial suspension (10^8^ CFU/ml) was added to the 4.5 ml of TSB containing 500 μl of each water extract or ethanol extract of Uiseong garlic (500 mg/ml). PBS was used as a control group (A_0_). Uiseong garlic extracts were compared with polymyxin B (1 mg/ml) as a positive control and ampicillin (1 mg/ml) as a negative control since *A. hydrophila* exhibited resistance to ampicillin. Afterward, the mixture of Uiseong garlic extract with bacterial suspension was incubated at 37°C with 110 rpm. The bacterial absorbance was measured at 600 nm at every 0, 1, 2, 4, and 6 h, and the antibacterial activity was calculated using the following equation [24, 25]:

$$\text{Antibacterial activity (U)}=\sqrt{\frac{A_{0}-A}{A}}$$

(A_0_: control group absorbance ; A: tested group absorbance).

### Intracellular Protein Leakages of *A. hydrophila* after Treatment with Uiseong Garlic Extracts

The leakages of intracellular proteins from *A. hydrophila* were measured after treatment with Uiseong garlic extracts, according to Wang *et al*. (2015) with some modifications. An aliquot of 500 μl of *A. hydrophila* suspension (10^9^ CFU/ml) was added to 4.5 ml of TSB containing 500 μl water and ethanol extract of Uiseong garlic, respectively. The suspensions were then incubated at 37°C with agitation at 110 rpm. Samples were taken at every 0, 1, 2, 4, and 6 h and then centrifuged at 7,000 ×*g* for 4 min. Subsequently, the protein content of the collected bacterial cells was immediately measured using the Bradford method [26].

### Morphological Observation of A. hydrophilia after Treatment with Uiseong Garlic Extracts

The morphological changes of A. hydrophilia after treatment with water or ethanol extract of Uiseong garlic were observed. A. hydrophilia suspension (10^8^ CFU/ml) was treated with water or ethanol extract of Uiseong garlic, polymyxin B, and PBS as a negative control, then incubated at 37°C for 12 h. After incubation, the suspension was washed with PBS three times and centrifuged at 7,000 ×*g* for 4 min. The collected bacterial cells were then placed on a copper grid (200 mesh Cu, Ted Pella Inc., USA) and stained with 2% phosphotungstic acid (Sigma-Aldrich Co.) for 30 sec. The images of bacterial cells were observed with transmission electron microscopy (TEM; HT7700, Hitachi Ltd., Japan) at 5000x magnification [27].

### Statistical Analysis

All experiments were conducted in triplicates. The data analysis was performed using the SAS (statistical analysis system) program (version 8.0, SAS Institute, SA). Comparisons between the groups or treatment were conducted using *t*-test, one-way analysis of variance (ANOVA), and Duncan’s multiple range test. A *p*-value of less than 0.05 was considered to be statistically significant.

## Results

### Comparison of Yields

Uiseong garlic was extracted with water and ethanol at 22°C and 90°C, respectively. As shown in Table 1, the yield of water extracts was significantly greater than that of ethanol extracts (*p* < 0.05). The water extract at 90°C had the highest yield (25.15±1.56%), followed by the water extract at 22°C (20.59±0.01%), the ethanol extract at 90°C (4.32±0.07%), and the ethanol extract at 22°C (2.55±1.30%). The higher the temperature, the higher yield was extracted from water and ethanol.

### Antibacterial Activities of Uiseong Garlic Extracts

The antibacterial activities of Uiseong garlic extracts were investigated against 22 foodborne pathogens (Table 2). Ethanol extract at 22°C showed antibacterial activities against all foodborne pathogens, including nine strains of A hydrophila. In contrast, water extract at 22°C had antibacterial activities against 14 foodborne pathogens. Among these bacteria, ethanol extract at 22°C showed the highest antibacterial activity against *A. hydrophila* SNUFPC A7 (18.33±0.58 mm). In addition, ethanol extract at 22°C exhibited significantly greater (*p* < 0.05) antibacterial activities than water extract at 22°C except for *Salmonella* Enteritidis and *Shigella sonnei*. However, extracts at 90°C did not exhibit any antibacterial activities against whole bacterial strains. Therefore, Uiseong garlic extract at 22°C was selected to evaluate their mechanism and antibacterial activities against *A. hydrophila*.

### Minimum Inhibitory Concentration of Uiseong Garlic Extracts

The MIC of Uiseong garlic extracts at 22°C for nine strains of *Aeromonas* spp. are shown in Table 3. Both water and ethanol extract at 22°C exhibited the most potent antibacterial activities with a MIC value of 10 mg/disc against *A. hydrophila* JUNAH, *A. hydrophila* SNUFPC A3, *A. hydrophila* SNUFPC A7, *A. hydrophila* SNUFPC A8, and *A. hydrophila* SNUFPC A9. Furthermore, the largest clear zone diameter indicated the highest antibacterial activity was observed against *A. hydrophila* SNUFPC A7 for both water and ethanol extract at 22°C with the size of 11.50±0.71 mm and 10.00±0.00 mm, respectively. Based on this result, strain *A. hydrophila* SNUFPC A7 was selected as a target to identify the antibacterial mechanism of Uiseong garlic extracts.

### Antibacterial Dynamics of Uiseong Garlic Extracts

**Fig. 1:**
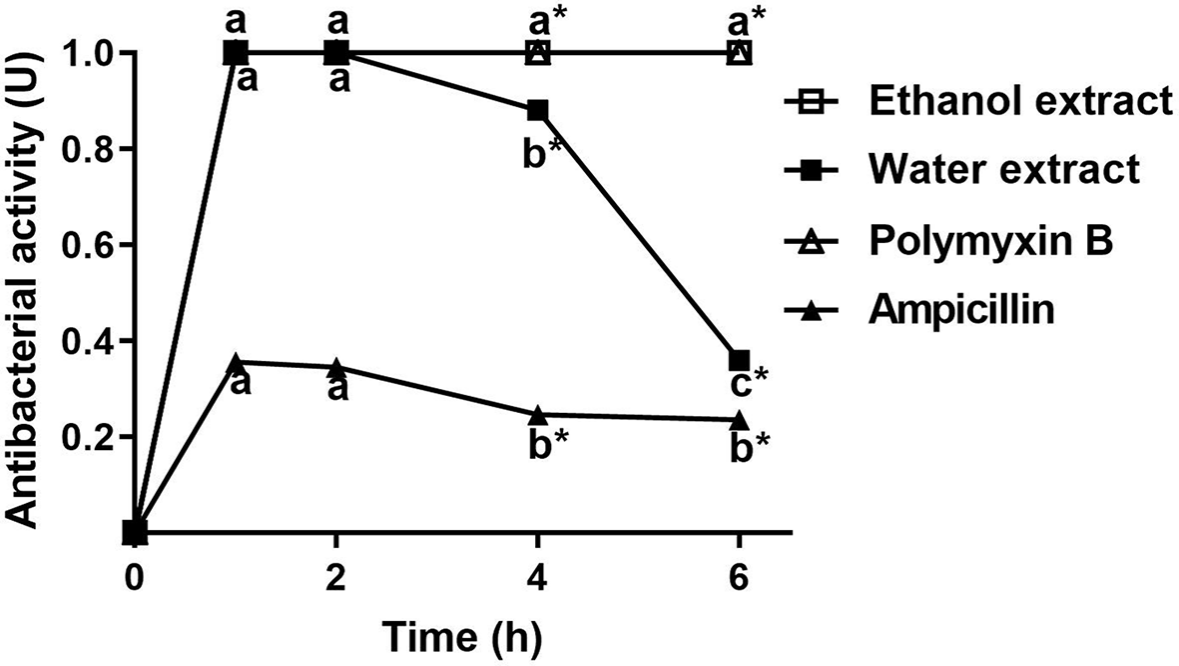
The antibacterial dynamics of Uiseong garlic extracts against *A. hydrophila* SNUFPC A7. Concentration of water and ethanol extract: 500 mg/ml, polymyxin B and ampicillin: 1 mg/ml. Asterisks (*) indicate significant differences between treatments at the same incubation time (*p* < 0.05). Different letters (a-c) indicate significant differences between incubation times in the same group (*p* < 0.05).

The relationship between the antibacterial activity and incubation time is presented in Fig. 1. Due to the antibiotic resistance of *A. hydrophila* SNUFPC A7, ampicillin exhibited significantly lower antibacterial activity (0.24 0.00 to 0.36 0.06 U) compared to other treatments (*p* < 0.05). Antibacterial activities of water extract, ethanol extract, and polymyxin B increased sharply within the first hour. In the whole incubation time, the ethanol extract exhibited the same pattern of antibacterial dynamics with polymyxin B, and there was no significant difference between the two groups (*p* < 0.05). In addition, from 1 h of incubation, both ethanol extract and polymyxin B exhibited stable antibacterial activity at 1.00 0.00 U. However, the antibacterial activity of water extract peaked at two hours before significantly decreasing to 0.36 0.00 U by 4 h. Significant differences in antibacterial activity between the water extract and ethanol extract were observed at incubation times of 4 and 6 h (*p* < 0.05).

### Protein Leakages of *A. hydrophila* SNUFPC A7 by Uiseong Garlic Extracts

**Fig. 2:**
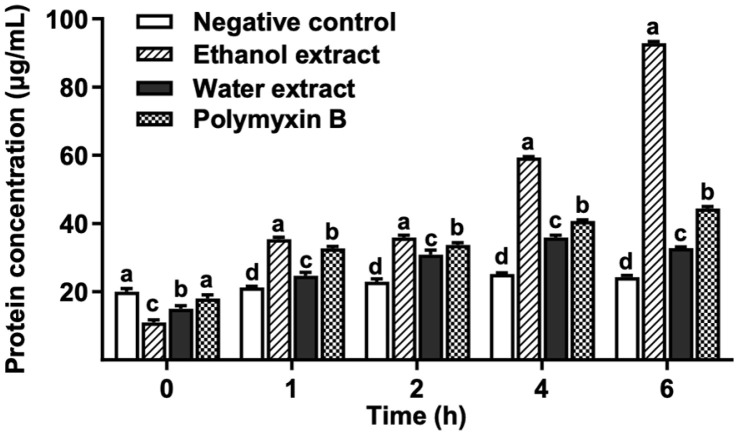
The leakage of intracellular proteins of *A. hydrophila* SNUFPC A7. Concentration of water and ethanol extract: 500 mg/ml, polymyxin B: 1 mg/ml. Different letters (a-d) indicate significant differences between treatments in the same incubation time (*p* > 0.05).

The pattern of protein leakages in *A. hydrophila* SNUFPC A7 were analyzed following the addition of Uiseong garlic extracts, with the concentration of leaked proteins compared for 6 h of incubation (Fig. 2). The leakage of proteins in bacteria was increased significantly as the incubation time increased (*p* < 0.05). At initial incubation time, the concentration of leaked proteins induced by Uiseong garlic extracts were significantly lower than negative control and polymyxin B (*p* < 0.05). However, after 1 h, both Uiseong garlic extracts induced significantly higher protein leakages than the negative control (*p* < 0.05). Furthermore, protein leakages induced by the ethanol extract were significantly higher than the water extract throughout the entire incubation period (*p* < 0.05). Additionally, in comparison between ethanol extract and polymyxin B, the concentration of protein leakages induced by the ethanol extract was significantly higher than those treated with polymyxin B after 1 h of incubation (*p* < 0.05). In addition, ethanol extract exhibited the highest concentration of leaked protein (92.87±0.46 μg/ml) at 6 h of incubation time. Consequently, the ethanol extract from Uiseong garlic exhibited greater antibacterial activities targeting the cell membrane compared to both water extract and polymyxin B.

### Morphological Changes of *A. hydrophila* SNUFPC A7 by Uiseong Garlic Extracts

**Fig. 3:**
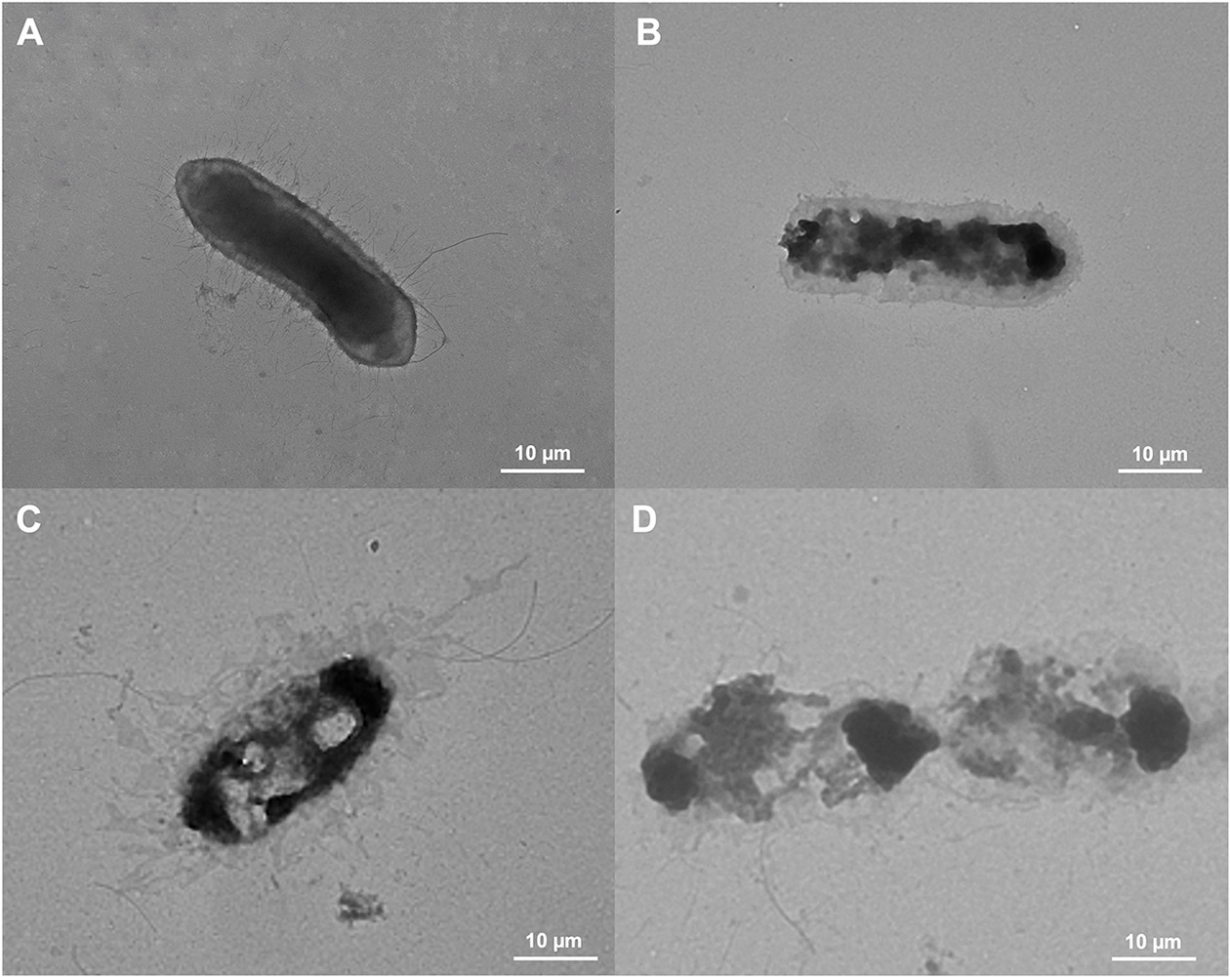
TEM images of *A. hydrophila* SNUFPC A7 after treatment with (A) PBS (negative control), (B) water extract of Uiseong garlic (500 mg/ml), (C) ethanol extract of Uiseong garlic (500 mg/ml), and (D) polymyxin B (1 mg/ml) at 5,000× magnification.

TEM was used to observe the morphological changes in *A. hydrophila* SNUFPC A7 after treatment with Uiseong garlic extracts (Fig. 3). The negative control exhibited a smooth and ordinary surface without any ruptures or pores on the cell membrane (Fig. 3A). However, treatment with water extract, ethanol extract, and polymyxin B exhibited noticeable changes in the cell membrane due to the presumably outflow of cell constituents (also known as plasmolysis) (Fig. 3B-3D). These noticeable changes in the cell membrane increased the extent of damage, demonstrating that both Uiseong garlic extracts exhibited antibacterial activities against *A. hydrophila* SNUFPC A7.

## Discussion

The aquatic environment often serves as a medium for accumulating and spreading antibiotic-resistant bacteria because they are often closely associated with human activities [28]. In this context, the use of garlic extracts could provide a valuable strategy for controlling *A. hydrophila* in aquatic environments. In the present study, Uiseong garlic was extracted using water and ethanol at both temperatures of 22 and 90°C. The extraction of Uiseong garlic using water as solvent resulted in approximately 8-fold and 6-fold higher yield, at 22 and 90°C, respectively, compared to ethanol extraction (*p* < 0.05). This result can be attributed to the polar nature of water, which may facilitate the extraction of a broader range of polar compounds present in garlic, including polysaccharides and glycosides [29]. Park & Chin [13] also reported a similar result in which water extraction of garlic resulted in approximately 13-fold higher yield than methanol extraction [13]. Moreover, the significantly greater yields of Uiseong garlic extract at 90°C implied that high temperature may enhance the efficiency of the extraction process, presumably by promoting the disruption of cellular structures and facilitating the release of bioactive compounds [30].

Our study demonstrated the broad antimicrobial activities of ethanol extract of Uiseong garlic at 22°C against all tested foodborne and spoilage bacteria, whereas the water extract at 22°C did not exhibit antimicrobial activities against *Bacillus* spp., *E. coli*., *L. monocytogenes*, *S. aureus*, and *Vibrio parahaemolyticus*. These findings indicated that the ethanol extract was more effective than the water extract in inhibiting bacterial growth. Consistent with our results, Fujisawa *et al*. [31] reported that garlic extracted with ethanol contained a higher concentration of allicin compared to water extraction, leading to greater antibacterial activity. These higher activities may be attributed to the hydrophobic nature of organosulfur compounds in garlic, which dissolves better in alcohol than in water. Moreover, ethanol contains a hydroxyl group that can help to stabilize these compounds, enhancing their antimicrobial effectiveness. The lower antibacterial activity observed in water extracts may be due to the lower solubility of organosulfur compounds and other antimicrobial agents in water. Therefore, ethanolic solutions can be considered more effective than water in extracting antibacterial compounds from garlic [31]. However, despite higher yields, Uiseong garlic water and ethanol extracts at 90°C did not exhibit any antibacterial activities against all tested foodborne and spoilage bacteria in this study. This result agreed with previous studies by [16, 32], which showed that antibacterial activities of garlic extracts might be disrupted during high temperatures (80-90°C) heating, possibly due to physical or chemical alteration and volatilization of the antibacterial components [16, 32].

Due to the emergence of bacteria resistant to the most common-use antibiotic, polymyxins have become the last line of defense against multiple drug-resistant Gram-negative bacteria infections, including *A. hydrophila* [1, 28]. Polymyxin carries a net polycationic charge and interacts with the negatively charged phosphate groups of the lipid A from bacterial lipopolysaccharide (LPS) [1]. Additionally, its hydrophobic fatty acyl side chains bind to corresponding LPS structures, leading to the disruption of bacterial cell walls and cell death [33, 34]. In the present study, Uiseong garlic extracts were compared with polymyxin B in terms of antibacterial dynamic, mechanisms of action, and change in bacterial morphology against *A. hydrophila* SNUFPC A7.

To further explore the antibacterial mechanism of Uiseong garlic water and ethanol extracts, the antimicrobial dynamics were conducted to compare the antibacterial activities with polymyxin B over six hours (Fig. 1). Similar patterns of antibacterial dynamics were observed between ethanol extract and polymyxin B, suggesting a comparable antibacterial efficiency. This finding indicated that the ethanol extract of Uiseong garlic had a strong antibacterial activity, presumably due to its high composition of organosulfur compounds [14]. The results of our study align with the findings of Yoo *et al*. [14], which demonstrated that Uiseong garlic contains significantly two times higher concentrations of organosulfur compounds, including alliin (26.31 mg/g), allicin (7.47 mg/g), γ-glutamyl-S-allyl-L-cysteine (GSAC) (13.17 mg/g), and γ-glutamyl-S-trans-1-propenyl-L-cysteine (GSPC) (38.87 mg/g) compared to other garlic varieties harvested in different areas (Seosan, Danyang). The high levels of organosulfur compounds in Uiseong garlic reinforce its potential as a natural antibacterial agent and indicate that its strong antibacterial activity is primarily due to its unique biochemical profile.

The protein leakages observation (Fig. 2) also revealed that the ethanol extract of Uiseong garlic disrupted the highest amount of *A. hydrophila* SNUFPC A7 proteins even after 6 h of exposure. This result suggests that the antibacterial mechanism of garlic extract likely involves disruption of the bacterial cell membrane. This membrane disruption leads to the loss of essential intracellular contents [35]. Conversely, the water extract of garlic exhibited lower antibacterial activity compared to ethanol extract and polymyxin B. The decline in antibacterial activity after two hours may be attributed to the difference in the solubility and bioavailability of antimicrobial constituents in water compared to ethanol extract [36]. Nonetheless, the water extract showed protein leakage from *A. hydrophila* SNUFPC A7, indicating potential disruption of cell membrane integrity and cellular function [35]. Moreover, TEM analysis (Fig. 3) revealed the presence of gaps in the bacterial cell membrane after treatment with Uiseong garlic extracts and polymyxin B, indicating the formation of pores. These pores compromise cell membrane integrity, leading to a loss of cell viability [35]. This result indicated that both treatments with Uiseong garlic extracts damaged the cell wall, which increased the permeability of the bacterial cell membrane [25].

The study confirmed that Uiseong garlic possessed broad antimicrobial activities, mainly when extracted with ethanol at lower temperatures. Comparative analyses between Uiseong garlic extracts and polymyxin B, showed that the ethanol extract had a comparable antibacterial efficiency against *A. hydrophila*. These findings highlight the potential of ethanol extract of Uiseong garlic as a natural alternative to combat *A. hydrophila*. However, further investigation related to the specific bioactive compounds responsible, their modes of action, and the cytotoxicity of the extract is needed to ensure its efficacy and safety in practical applications.

## Figures and Tables

**Table 1:** Yield (%) of Uiseong garlic extract in different temperatures and solvents.

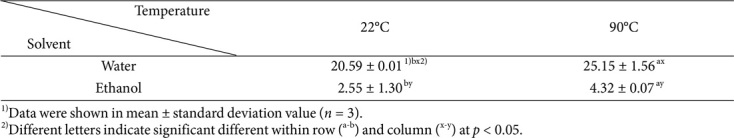

**Table 2:** Antibacterial activities (mm) of Uiseong garlic extracts against foodborne pathogens.

Bacterial strains	22°C		90°C		Positive control		Negative control
	Water extract	Ethanol extract	Water extract	Ethanol extract	Ampicillin	Polymyxin B	Distilled water
*Aeromonas hydrophila* ATCC 7966	11.33±0.58 ^1)Cy2)^	14.33±0.58 ^EFGx^	-^3)^	-	-	12.67±0.58	-
*A. hydrophila* JUNAH	13.00±1.00 ^By^	17.00±1.00 ^BCx^	-	-	-	13.33±0.58	-
*A. hydrophila* SNUFPC A3	13.00±1.00 ^By^	15.33±0.58 ^DEFx^	-	-	-	13.00±0.00	-
*A. hydrophila* SNUFPC A5	14.33±0.58 ^ABy^	16.67±0.58 ^BCDx^	-	-	-	11.67±0.58	-
*A. hydrophila* SNUFPC A7	14.67±0.58 ^Ay^	18.33±0.58 ^Ax^	-	-	-	12.67±0.58	-
*A. hydrophila* SNUFPC A8	13.67±0.58 ^ABy^	17.67±0.58 ^ABx^	-	-	-	13.00±0.00	-
*A. hydrophila* SNUFPC A9	14.33±1.15 ^ABy^	16.67±0.58 ^BCDx^	-	-	-	14.00±1.00	-
*A. hydrophila* SNUFPC A10	13.00±1.00 ^By^	16.67±0.58 ^BCDx^	-	-	-	12.33±0.58	-
*A. hydrophila* SNUFPC A11	9.67±0.58 ^Dy^	13.67±0.58 ^GHIx^	-	-	-	12.67±0.58	-
*Bacillus cereus* ATCC 13061	-	12.33±0.58 ^IJ^	-	-	17.50±0.71	10.50±0.71	-
*B. subtilis* ATCC 6633	-	15.67±0.58 ^CDE^	-	-	-	10.50±0.71	-
*Escherichia coli* O157:H7 ATCC 10536	-	9.33±0.58 ^M^	-	-	18.00±0.00	10.50±0.71	-
*E. coli* ATCC BAA02192	-	9.21±0.05^M^	-	-	17.50±0.20	10.50±0.50	-
*Listeria monocytogenes* ATCC 7644	-	13.33±1.53 ^GHI^	-	-	19.50±0.71	11.00±0.00	-
*L. monocytogenes* ATCC 19115	-	13.50±0.58^GHI^	-	-	19.00±0.00	11.00±0.00	-
*Pseudomonas aeruginosa* ATCC 9027	9.67±0.58 ^Dy^	13.00±0.00 ^GHIx^	-	-	19.00±0.00	11.50±0.71	-
*P. aeruginosa* ATCC 10145	9.65±0.58^Dy^	13.20±0.02 ^GHIx^	-	-	19.00±0.00	11.50±0.71	-
*Salmonella* Enteritidis ATCC 13076	9.67±0.48 ^Dx^	9.67±0.58 ^LMx^	-	-	17.50±0.71	11.00±0.00	-
*Shigella sonnei* ATCC 9290	10.67±0.58 ^CDx^	11.33±0.58 ^JKx^	-	-	19.00±1.41	11.00±0.00	-
*Staphylococcus aureus* ATCC 25923	-	10.67±1.15 ^KL^	-	-	19.00±0.00	10.50±0.71	-
*Vibrio parahaemolyticus* ATCC 17802	-	14.33±0.58 ^EFG^	-	-	19.50±0.71	11.50±0.71	-
*Yersinia enterocolitica* ATCC 23715	10.33±0.58 ^CDy^	14.00±1.00 ^FGHx^	-	-	10.00±0.00	12.00±0.00	-

^1)^Data were shown in mean±standard deviation value (*n* = 3).

^2)^Different letters indicate significant different within row (^x-z^) and column (^A-M^) at *p* < 0.05.

^3)^Not detected.

**Table 3:** Minimum inhibitory concentration of Uiseong garlic extracts at 22°C against *A. hydrophila*.

Bacterial strains	Water extract		Ethanol extract	
	MIC (mg/disc)	Inhibition zone (mm)	MIC (mg/disc)	Inhibition zone (mm)
*Aeromonas hydrophila* ATCC 7966	20	10.500.71^1)^	20	9.000.00
*A. hydrophila* JUNAH	10	11.000.00	10	9.500.71
*A. hydrophila* SNUFPC A3	10	10.000.00	10	9.000.00
*A. hydrophila* SNUFPC A5	20	10.000.00	10	9.000.00
*A. hydrophila* SNUFPC A7	10	11.500.71	10	10.000.00
*A. hydrophila* SNUFPC A8	10	11.000.00	10	9.500.71
*A. hydrophila* SNUFPC A9	10	10.500.71	10	9.000.00
*A. hydrophila* SNUFPC A10	15	10.500.71	15	9.000.00
*A. hydrophila* SNUFPC A11	15	9.500.71	15	9.500.71

^1)^Data were shown in mean±standard deviation value.
